# Preoperative Fasting among Adult Patients for Elective Surgery in a Kenyan Referral Hospital

**DOI:** 10.1155/2017/2159606

**Published:** 2017-04-12

**Authors:** George Njoroge, Lucy Kivuti-Bitok, Samuel Kimani

**Affiliations:** ^1^School of Nursing Sciences, University of Nairobi, P.O. Box 19676, Nairobi 00202, Kenya; ^2^Kenyatta National Hospital, P.O. Box 20723, Nairobi 00202, Kenya

## Abstract

*Background*. Preoperative fasting (POF) is physiologically and precautionary important during anesthesia and surgery. POF from midnight has been practiced despite the recommended shorter practice.* Objective*. Assessing preoperative fasting among adult patients scheduled for elective surgery at Kenyatta National Hospital (KNH).* Methods*. A descriptive cross-sectional study involving 65 surgical patients. A questionnaire of mixed questions on demographics, reasons, source of instructions, opinion on instructions, time, premedication practices, outcome, and complains on NPO was used. Analysis was quantitatively done with SPSS v. 22. Ethical approval was obtained from KNH-UoN ERC.* Results*. Of the respondents 93.8% lacked knowledge on the correct reasons for POF and felt that the instructions were unclear and less important <50%. POF instructions were administered by nurses 80%, anesthetists 15%, and surgeons 5%. Most of respondents (73.8%) fasted > 15 hours. The POF outcomes were rated moderately challenging as follows: prolonged wait for surgery 44.6%, thirst 43.1%, hunger 36.9%, and anxiety 29.2%.* Conclusion*. Nurses are critical in providing POF instructions and care, and patient knowledge level is a mirror reflection of the quality of interventions. This underscores the need to build capacity for nurses and strengthen the health system to offer individualized preoperative interventions as well as monitoring and clinical auditing of fasting practices.

## 1. Introduction

Preoperative fasting (POF) is a time tested professional practice that is undertaken for physiological and precautionary benefits to the patients globally. Patients are deprived of certain and/or all foods and drinks for specific duration before surgery [1]. For clinical purposes, POF is abstinence from all foods and liquids for a specified period of time before induction of anesthesia and/or commencement of surgery. The duration for POF is dictated by the type of diet, patient condition, and the kind of surgery whether emergency or elective among other factors. Some diets, regardless of their texture (solids or fluids), are easily digestible, thus allowing for rapid elimination from the stomach, while others are slow release type stagnating in the gastrointestinal tract [2]. The POF provides empty or near empty stomach, a critical requirement for emergency, and elective surgical interventions [3]. The POF intervention ensures physiological stability, reduction of complications, hospital stay, and costs [4]. The practice is structured to include well-planned and packaged health information on the goal and expectations for the patient thus, promoting compliance and allaying anxiety.

Physiologically, the goal of POF is to minimize the risk of regurgitation, vomiting, aspiration, and the complications thereof during anesthesia or surgery [5]. The prescription and execution of POF are majorly standardized, spanning from 2 to 8 hours based on the aforementioned factors [2, 3]. The patients who report drinking clear liquids, nonhuman milk, and light and/or regular meals are fasted for 2, 6, and 8 hours, respectively [2]. The gold standard for the specific POF duration for a patient fed on liquid or solid diet has been prescribed and adopted globally [6]. The guidelines are flexible, and they prescribe safe and friendlier package to the patient, with no significant complications reported in their implementation [7]. The prescribed POF guideline is a departure from traditional practice where patients were fasted from midnight to the following day's surgery regardless of the patient's condition, work load, theatre schedules, emergencies, and other logistical challenges. Despite the aforementioned advantages of the revised globally acceptable guidelines, some hospitals have maintained the time honored prolonged preoperative fasting practice notably nil per oral (NPO) from midnight [6].

The instructions (health messages) for POF are issued by clinical team members, namely, nurses, physicians, anesthetists, or surgeons [6]. The instructions should be clear including the objective of POF, duration, and the expectations as well as the consequences of nonadherence. Therefore, the clinician should be conversant with the guidelines and communicate and deliver clear instructions to the patients [7]. Lack of knowledge, rigidity, and/or poor strategies for disseminating fasting guidelines among the clinical team impede patient understanding and fasting compliance [8, 9]. The lack of knowledge has also contributed to the persistence of old practices in the health care systems [10, 11]. There are other varied reasons for the persistence of the practice including work overload, insufficient time for individualized patient instructions, and resistance to change [12]. The situation portends that patient fasts more than the stipulated duration [13]. This is particularly common among patients scheduled for surgeries carried out in the afternoon. Thus, for such patients instead of NPO from midnight, the POF duration should be guided by the last meal or the fluid taken. Additionally, patients with comorbidities, for example, diabetes and hypertension, are allowed taking their medication together with liquids amounting to 150 mls an hour before surgery [14]. The revised guidelines provide that consumption of meals or liquids close to time of surgery guided by the fasting guidelines could be helpful, contrary to fear of the presence of food or liquid in the stomach among many clinicians. Indeed, the fear of regurgitation has been implicated in prolonged fasting and its consequences [15, 16].

A shortened POF time based on evidence based guidelines is beneficial to the patient because it increases postoperative comfort, improves insulin resistance, and reduces stress responses [17]. However, the adoption of the reviewed POF guidelines and their attendant benefits has been inconsistent globally. There are professionals in both developed and developing countries that are not following these guidelines [18–20] particularly in terms of adherence to fasting time and instructions, leading to excessive fasting.

Locally, at Kenyatta National Hospital (KNH), a referral and teaching hospital in Kenya, the time honored fasting practice has persisted (anecdotal evidence). However, the persistence and inflexibility in adopting the universally accepted reviewed practice have not been investigated so far [21, 22]. This study therefore assessed the preoperative fasting among adult patients scheduled for elective surgery at KNH.

## 2. Methods

This was a descriptive cross-sectional study involving purposively selected respondents (*n* = 65) from surgical wards at KNH conducted between April and July 2015. The sample size calculation was based on a formula by O. Mugenda and A. Mugenda [23]. During the study duration, the hospital theatres operated on 3332 patients with surgical problems. However, after excluding the pediatric, obstetric, unconsenting subjects and those who were operated outside 8 a.m. to 5 p.m. and weekends, an estimated total of 100 patients were established. Therefore from the estimate, the sample size was established as 79 subjects who were presented with the questionnaires. From the sample, a total of 65 subjects completed the questionnaire representing a response rate of 82%.

Data was collected using a self-administered structured questionnaire developed by the researchers. The questionnaire was structured into closed ended questions for the quantitative data, as well as open-ended ones for the qualitative data. The components in the questionnaire included demographic characteristics, reasons for POF, complains about NPO, source of POF instructions, opinion on POF instructions, POF time, premedication practices, and outcome of prolonged POF, respectively.

The questionnaire was administered 30 minutes before the premedication; thereafter a follow-up to ascertain the exact time of the commencement of surgery was done. Data was collected using a self-administered questionnaire among the literate respondents, while those with literacy challenges were helped in reading and filling the questionnaires by trained research assistants. The quantitative data was screened, coded, and entered into the analytical computer software (SPSS v. 22) and analyzed. The descriptive and inferential statistics were generated and reported accordingly. Specifically, the mean, mode, median, standard deviation and chi-squares were generated and discussed. The qualitative data generated from the open-ended questions was screened and organized and repeating themes were grouped together. The most common repeating themes were manually identified, captured, and the quotes have been included in the results.

The ethical approval to conduct the study was obtained from KNH-University of Nairobi Ethical Review Committee (KNH-UoN ERC) (Approval number P104/02/2015). The institutional permission was granted by the surgical departmental head (Ref. KNH/SAD SURG/15/VOL./63). Consent was obtained from the subjects both verbally and in written form before data collection.

## 3. Results

### 3.1. Demographic Characteristics of the Respondents

Demographically, the respondents were female (55.4%) and married (78.5%). The respondents had attained some formal education, namely, primary (33.8%), secondary (46.2%), college (13.8%), and university (6.2%). There was high (78.5%) unemployment rate among the respondents (Table 1).

### 3.2. Association between Demographic Characteristics and Complain about Nil per Oral among the Respondents

Respondents with low educational level reported more complains about nil per oral instructions compared to the other categories. Further analyses with chi-square showed a significant (*χ*^2^ = 8.473, df = 3, and *p* = 0.037) association between the level of education and complains about NPO. Respondents of low education level were likely to complain of NPO instructions (Table 2).

### 3.3. Knowledge on Preoperative Fasting among the Respondents

**Figure 1:**
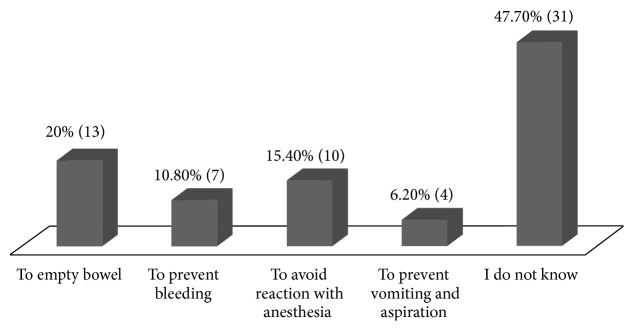
*Knowledge on preoperative fasting among respondents.* There was lack of knowledge regarding the reasons for preoperative fasting among the respondents. A majority (93.8%) gave incorrect reasons.

The study respondents demonstrated knowledge deficit regarding the reasons for preoperative fasting. Of the respondents, only 6.2% gave the correct reason for preoperative fasting, namely, prevention of vomiting and aspiration. Nearly a half of the respondents (47.7%) did not know the reason, while others gave irrelevant answers such as reaction to anesthesia (15.4%), prevent bleeding (10.8%), and empty bowel (20%), respectively (Figure 1). On further analyses, there was no association between knowledge on preoperative fasting and opinion on fasting instructions (*χ*^2^ = 5.261, df = 4, and *p* = 0.262).

### 3.4. Source of Preoperative Fasting Instructions among Respondents

**Figure 2:**
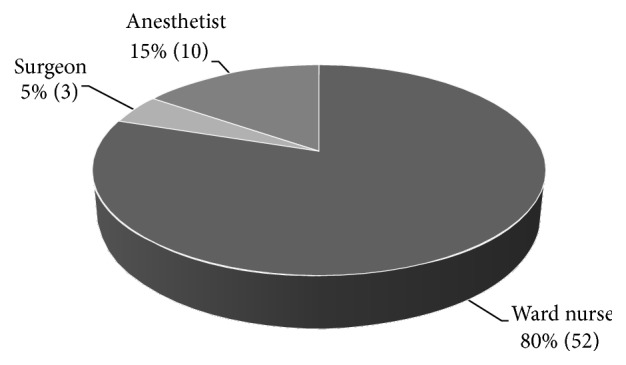
*Sources of preoperative fasting instructions among the respondents.* Preoperative fasting instructions were communicated by nurses (80%) to the respondents.

The respondents reported varied sources of POF instructions. The instructions were mainly delivered by the nurses (80%), anesthetists (15%), and the surgeons (5%), respectively (Figure 2). Further analyses showed that respondents who reported nurses as their source of preoperative instructions were likely (*χ*^2^ = 6.164, df = 2, *p* = 0.046) to complain about NPO.

### 3.5. Duration of Preoperative Fasting among the Respondents

**Figure 3:**
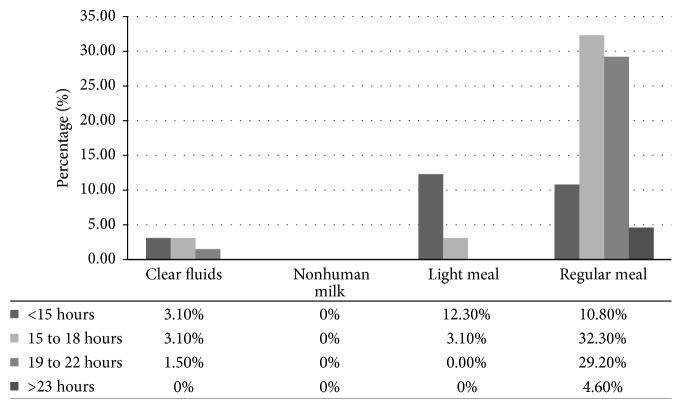
*Duration of preoperative fasting among the respondents.* A majority (73.8%) of respondents fasted for more than 15 hours.

The respondents reported consuming various types of meals before POF including clear fluids, nonhuman milk, and light or regular meal. None of the respondent reported consumption of nonhuman milk (cow's milk, soya milk, or rice milk) meal (Figure 3). Of the ones (7.7%) who consumed clear fluids, 3.1% fasted for less than 15 hours, while 3.1% starved for 15 to 18 hours and 1.5% fasted between 19 to 22 hours. Among the respondents (15.4%) who consumed light meal, 12.3% fasted for 15 hours or less, while 3.1% starved for 15 to 18 hours.

A majority (76.9%) of the respondents consumed regular meals; of them 10.8% fasted for less than 15 hours, while 32.3% starved between 15 and 18 hours, whereas 29.2% did not eat for between 19 and 22 hours, while 4.6% starved for more than 23 hours (Figure 3). Further analyses did not yield any statistical significance between knowledge on preoperative fasting and fasting practice after consumption of meals (*χ*^2^ = 1.763, df = 4, and *p* = 0.779).

### 3.6. Perception on Preoperative Fasting Instructions among the Respondents

**Figure 4:**
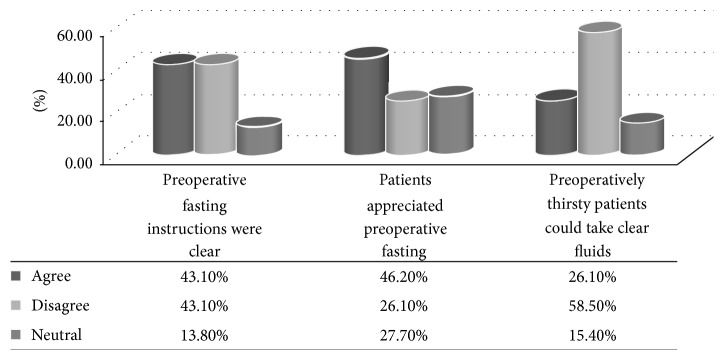
*Perceptions on preoperative fasting instructions among respondents.* Respondents felt that the preoperative instructions were unclear (43.1%) and less important (46.2%).

The respondents displayed varied perceptions regarding preoperative fasting instructions. Less than half (43.1%) of the respondents felt that the instructions were clear, while 43.1% viewed them to be unclear. However, only 13.8% did not support either of the responses. Of the respondents, 46.2% appreciated the critical role of the instructions, while 26.1% felt that they were less important. However, 27.7% were noncommittal regarding the importance of POF. On whether clear fluids could be taken by a thirsty patient while awaiting surgical intervention, only 26.1% (*n* = 17) supported the view and 58.5% (*n* = 38) did not, while 15.4% (*n* = 10) were indifferent (Figure 4).

### 3.7. Knowledge on Ingestion of Medication during Fasting among the Respondents

Regarding ingestion of medication during fasting, the majority (72.3%) supported the idea, while 28% reported that medication cannot be taken with clear fluids. These findings were corroborated by the following excerpts from the respondents on preoperative medications. P003…* “It helps in drug absorption, can take antihypertensive with little fluid”*…; P009 …*“To reduce hunger”…*; P017 …*“To reduce thirst”…; *P031…*“It was part of eating”…*; P042 …* “Should not take any food before surgery”…*; and P048* “*…*Told not to take anything by mouth”*….

### 3.8. Outcomes on Preoperative Fasting among the Respondents

**Figure 5:**
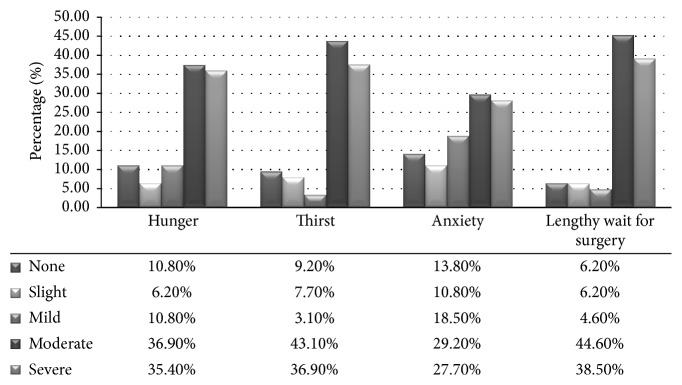
*Outcomes of preoperative fasting among respondents.* The preoperative fasting outcomes were rated moderately challenging by the respondents including prolonged wait for surgery (44.6%), thirst (43.1%), hunger (36.9%), and anxiety (29.2%), respectively.

The respondents reported various POF outcomes, for example, hunger, thirst, anxiety, and prolonged wait for surgery. On ascending order of their severity, the outcomes were grouped into none, slight, mild, moderate, and severe. Majority of the respondents ranked all the outcomes as moderately challenging as follows: hunger (36.9%), thirst (43.1%), anxiety (29.2%), and prolonged wait for surgery (44.6%), respectively (Figure 5). There was a significant (*χ*^2^ = 38.617, df = 16, *p* = 0.001) association between knowledge on consumption of fluid and perception of thirst among the respondents. Respondents who demonstrated low knowledge on consumption of fluid were likely to experience severe thirst.

## 4. Discussion

The findings of our study are summarized as follows: (i) adult patients scheduled for elective surgery demonstrated knowledge deficit regarding the reasons for preoperative fasting, (ii) the patients felt that the preoperative fasting instructions were not clear and less important, (iii) nurses were the main source of the preoperative instructions and the patients who received these instructions were likely to complain, (iv) patients scheduled for elective surgery reported being subjected to prolonged fasting beyond the recommended duration, and (v) the outcomes associated with POF were reported as moderately challenging by patients awaiting surgery. These findings on POF are not consistent with the reviewed and acceptable global guidelines particularly in regard to the duration of fasting [6]. This may be due to lack of flexibility and adaptability by the institution to the global trends and development. Additionally, there could be lack of updates and capacity enhancement regarding the fast changing practices in medicine and nursing practice. This state of the practice is disadvantageous to the patients and compromises on medical and nursing training, the core mandate of the institution under study with serious downstream effect on the national health care system.

There was knowledge deficit on the reasons for POF, as well as unclear and less important fasting instructions among the patients. This is a major hindrance to quality surgical care and a criticism to the health care system. The adherence to POF protocols depends on clear understanding of the communicated and shared instructions and the perception of their importance thus affecting patients' outcomes [24]. Knowledge deficit on the reasons for POF among patients scheduled for surgery has been reported elsewhere [25]. Additionally, knowledge deficits, poor attitude by patients, and care givers have been implicated in nonadherence to fasting protocols and failure to implement fasting guidelines compromising surgical care [6].

Nurses were the main source of communication and sharing of POF instructions. They form the bulk of health care workforce and spend the longest time with the patients. Thus, they frequently interact with patients and encounter opportunities during fasting phase where instructions are shared and communicated [26]. Traditionally, fasting instructions have been delivered through bulk (general) notably fasting for either morning or afternoon surgeries. These generalities of hospital protocols should be avoided because patients are unique individuals in their own rights, physiologically, and in many other ways. Such practices are probably because of workload and shortage of nursing staff, thus adopting task nursing modality. Whereas this model may deliver on the services, the possibilities of compromising the quality of care are very high. The approach should be relooked and instead replaced with individualized patient's instructions/care. As care givers, communicating and sharing instructions with patients on POF, are critical, thus the care giver must be aware of the revised fasting guidelines [27].

The POF instructions were reported by patients to be unclear and less important. The commencement of POF was scheduled after lunch or evening meals, while others after midnight constitute a miscommunication according to the revised guidelines. The findings are consistent with reports that showed that patients fasted for long hours before surgery because they did not understand the shared POF instructions [28, 29]. The old ritualistic POF instructions from midnight resulted in prolonged fasting. At midnight when fasting instructions are communicated, some patients have already fasted longer. The care givers should therefore explain to the patients the importance and how long they should fast guided by the type of diet consumed. Clear instructions promote compliance and adherence to the interventions. Therefore, to avoid prolonged fasting, care givers should share fasting instructions with rationale to promote compliance [30]. The instructions should be communicated orally as well as in documentation with a copy filed in the patients information file for reference purposes [16]. Similarly, a written copy of the POF instructions should be given to the patient relatives for their reference and reinforcement. This is critical because relatives may interfere with the fasting programs especially in institutions where supplementary foodstuffs are not prohibited.

The patient for elective surgery reported that they could not consume clear fluids when experiencing thirst. This finding portends lack of knowledge and the inconsistencies associated with the fasting guidelines, since clear liquids are permissible up to two hours before surgery [31–33]. Of importance, the patients reported that they could take antihypertensive medication with clear fluids before surgery. This is an indication of relevant knowledge that may have been communicated by their physician regarding management of hypertension and surgery. The patients were knowledgeable on control of hypertension in the presence of scheduled surgery, a finding corroborated elsewhere [34]. This signifies that clear instructions with rationale may result in improved understanding on the POF protocols and compliance [6, 8, 35, 36].

This study relied heavily on verbal reports on preoperative fasting of the patients, which may have introduced some recall bias and subjectivity. However, this challenge was circumvented through double questioning that enabled identification of any inconsistencies in the reports. The study involved patients who were awaiting surgical interventions, who may have been under some pain and anxiety, affecting response and consistency. This was mitigated through message sharing, clarification, reassurance, and psychosocial support for the patients. Furthermore, the participation to the study was purely voluntary and respondents were asked whether they were comfortable answering the questions and they were at liberty to discontinue. This ensured that their rights were respected and was in compliance with the ethical requirement for none coercion. Finally, our study involved a small sample size, purposively sampled which may affect its generalizability.

In conclusion, the bulk preoperative fasting instructions for adult patients scheduled for surgery is a clinical norm at KNH. Nurses are critical in providing preoperative fasting instructions and care, and the patient knowledge level is a mirror reflection of the quality of interventions. This underscores the need to build capacity for the care givers and strengthen the health system to respond to individualized preoperative interventions. Monitoring and clinical audits of fasting practices need to be instituted and enforced to detect and correct deviation as early as possible.

## Figures and Tables

**Table 1:** Demographic characteristics of respondents.

Characteristics	Frequency (*n*) (%)
Gender	
Male	29 (44.6)
Female	36 (55.4)
Marital status	
Married	51 (78.5)
Single	3 (4.6)
Divorced	9 (13.8)
Separated	2 (3.1)
Educational level	
Primary	22 (33.8)
Secondary/high	30 (46.2)
College/polytechnic	9 (13.8)
University	4 (6.2)
Employment status	
Employed	14 (21.5)
Unemployed	51 (78.5)

**Table 2:** Association between demographic characteristics and complain about nil per oral.

Characteristics	Complain about nil per oral		Level of significance
	Yes (%)	No (%)	
Gender			
Male	37.9	62.1	*χ* ^2^ = 2.675, df = 3, *p* = 0.102
Female	58.3	41.7	
Marital status			
Married	51.0	49.0	*χ* ^2^ = 1.338, df = 3, *p* = 0.720
Single	66.7	3.3	
Divorced	33.3	66.7	
Separated	50.0	50.0	
Educational level			
Primary	68.2	31.8	*χ* ^2^ = 8.473, df = 3, *p* = 0.037
Secondary/high	46.7	53.3	
College/polytechnic	11.1	88.9	
University	50.0	50.0	
Employment status			
Employed	28.6	71.4	*χ* ^2^ = 3.047, df = 1, *p* = 0.081
